# Off-target binding of ^18^F-MK-6240 tau PET imaging demonstrated by high spatial resolution dedicated brain PET scanner

**DOI:** 10.1007/s12149-026-02161-9

**Published:** 2026-02-02

**Authors:** Kazunari Ishii, Takahiro Yamada, Kohei Hanaoka, Hayato Kaida, Yasuyuki Kojita, Atsushi Kono, Kenji Ishii, Takashi Kato, Akinori Nakamura

**Affiliations:** 1 Department of Radiology, Kobe University Graduate School of Medicine, 7-5-2 Kusunoki-cho, Chuo-ku, Kobe, 650 − 0016 Hyogo Japan; 2 Department of Radiology, Kindai University Faculty of Medicine, Sakai, Osaka Japan; 3 Institute of Advanced Clinical Medicine, Kindai University Faculty of Medicine, Sakai, Osaka Japan; 4 Team for Neuroimaging Research, Tokyo Metropolitan Institute of Gerontology, Tokyo, Japan; 5 Department of Radiology, National Center for Geriatrics and Gerontology, Obu, Japan; 6 Department of Clinical and Experimental Neuroimaging, National Center for Geriatrics and Gerontology, Obu, Aichi Japan; 7 Department of Biomarker Research, National Center for Geriatrics and Gerontology, Obu, Aichi Japan

**Keywords:** Tau PET, Dedicated brain PET, High resolution, MK-6240

## Abstract

**Objective:**

To verify the regional distribution, especially off-target binding, of the tracer ^18^F-MK-6240, on tau PET images obtained with a high-resolution dedicated brain PET scanner.

**Methods:**

We retrospectively analyzed the ^18^F-MK-6240 PET data from subjects with cognitive decline who were enrolled in the BATON study. All scans were acquired using the same high-resolution brain-dedicated PET system. We calculated the standardized uptake value ratios (SUVRs) by using manually defined restricted cerebellar volumes of interest (VOIs) excluding meningeal spill-in, and we compared these SUVRs with those obtained using the conventional CenTauR cerebellar gray matter reference. We also quantified the off-target uptake in regions including the meninges, retina, and ethmoid sinus. Group comparisons and interaction effects were analyzed by *t*-tests and an ANOVA, and ROC analyses were performed to assess the diagnostic accuracy of each method.

**Results:**

Off-target accumulations were clearly observed in the substantia nigra, globus pallidum, and pineal gland. Higher extra-cerebral cortical uptake was observed in the meninges and centrum semiovale in tau-positive individuals, although these differences did not remain significant after a multiple comparison correction. The SUVRs derived with the use of restricted cerebellar VOIs were significantly higher than those from the CenTauR cerebellar VOIs in both the tau-positive and tau-negative groups (*p* < 0.01). A significant interaction between the diagnostic group and the reference VOI choice was detected (*p* = 0.019). The ROC analysis showed excellent diagnostic performance, with AUCs of 0.930 for CenTauR and 0.984 for the restricted reference VOIs (a nonsignificant difference).

**Conclusion:**

^18^F-MK-6240 PET imaging using a high-resolution brain-dedicated PET scanner revealed notable off-target binding and meningeal spill-in, which significantly affect quantification depending on the reference VOI selection. Optimizing reference regions by excluding extracerebral contamination enhances the reliability of tau PET analyses and may be crucial for longitudinal studies and multicenter trials.

## Introduction

First-generation tau positron emission tomography (PET) imaging has an off-target problem. Various second-generation tau PET imaging tracers are currently under development, and their clinical data have been verified. One of these new tracers, ^18^F-MK-6240, has shown excellent results, especially in the detection of three and four repeated tau proteins in Alzheimer disease (AD) [1]. A visual assessment method that provides both an overall assessment of brain tauopathy and the regional characterization of abnormal tau deposition has been proposed [2, 3].

An off-target issue concerning ^18^F-MK-6240 is its binding to the meninges, retina, and sinus [4, 5]. Tissot et al. also observed age-related binding of ^18^F-MK-6240 in the putamen and pallidum [6]. In addition, the accumulation of ^18^F-MK-6240 in the skull/meninges was observed to be significantly higher in females compared to males, whereas males showed higher accumulations in the superior parts of the cerebellum [4]. The uptake of ^18^F-MK-6240 in the pineal gland and substantia nigra has also been observed [7–10].

Concerning reference regions, Fu et al. reported that although the cerebellar gray matter is optimal for cross-sectional differentiation in studies using ^18^F-MK-6240, this tissue is susceptible to extracerebral signal contamination and high variability. In contrast, the pons and white matter offer greater stability for longitudinal analyses due to their lower uptake variability and contamination [11]. They also showed that when using cerebellar gray matter reference regions, eroding from both the inferior and superior sides may be necessary to account for extracerebral signal contamination. Extracerebellar signals are stable at the group level but seem to fluctuate between and within subjects, and they are physiologically unknown at this time. It is thus necessary to correct extracerebral signal contamination due to its high inter- and intra-subject variability [11].

We conducted the present study to investigate (*i*) the distribution of the off-target binding of ^18^F-MK-6240 and (*ii*) how spill-in accumulation from the meninges affects the tracer’s uptake in the cerebellum, which is used as a reference region. A brain-dedicated high-resolution PET scanner was used for these goals.

## Subjects and methods

### Subjects

This study was conducted as part of the BATON study, and eligible patients were selected from the BATON study data (https://jrct.mhlw.go.jp/en-latest-detail/jRCTs031210288) that were collected at Kindai University Hospital, where ^18^F-MK-6240 PET imaging was conducted with a BresTome brain-dedicated high-resolution PET scanner: (Shimadzu Corp., Kyoto, Japan) [12–17]. At the time of BATON study enrollment, the subjects gave their written informed consent for data collection and completed questionnaires approved by each participating site’s ethical committee. The present study adhered to the principles outlined in the Declaration of Helsinki and followed the Ethical Guidelines for Medical and Health Research Involving Human Subjects issued by Japan’s Ministry of Health, Labour, and Welfare.

The BresTome brain-dedicated PET scanner system offers time-of-flight (TOF) measurement with a silicon photomultiplier (SiPM) detector optimized for both head and breast examinations. The PET data were reconstructed using the algorithms and conditions with a list-mode dynamic row action maximum-likelihood algorithm, a subset of 100, a β-value of 100, a single iteration, a 240 ⋅ 240 matrix, a 264-mm transaxial field of view (FOV), and 1.1 mm/pixel. All of the present subjects underwent ^18^F-MK-6240-PET for tau imaging with the BresTome.

Magnetic resonance imaging (MRI) three-dimensional (3D)-T1 weighted images were scanned and produced using a Philips MRI scanner (Achieva 3.0T, Achieva dStream 1.5T). After 90 min of a 185 MBq dose of ^18^F-MK-6240 intravenous injection, a 20-min list-mode PET scan was acquired following the protocol of the Japan Agency for Medical Research and Development (AMED) study (jRCTsO31180219).

### Data analysis

Two nuclear medicine physicians independently interpreted the ^18^F-MK-6240 PET images to determine the presence or absence of tau deposition. Based on their assessments, the subjects were categorized into tau-positive and tau-negative groups following the visual interpretation algorithm for assessing brain tauopathy with ^18^F-MK-6240 PET [2, 3]. In the present study, cases with no ^18^F-MK-6240 accumulation in the cerebral cortex or accumulation that was limited to the entorhinal cortex only (Braak stage 0 or I) were defined as negative, while the cases with Braak stage II or higher were defined as positive. The off-target uptake inside and outside the brain parenchyma was also visually assessed in each case.

The anatomical normalization of the 3D-T1 weighted MR images (3D-MRI) and ^18^F-MK-6240 images was then performed using Statistical Parametric Mapping ver. 12 (SPM 12; Wellcome Department of Cognitive Neurology, London, UK). All individual ^18^F-MK-6240 images were co-registered to the individual 3D-MR images and then transformed into the standard stereotactic anatomical space using the DARTEL technique [18].

**Fig. 1:**
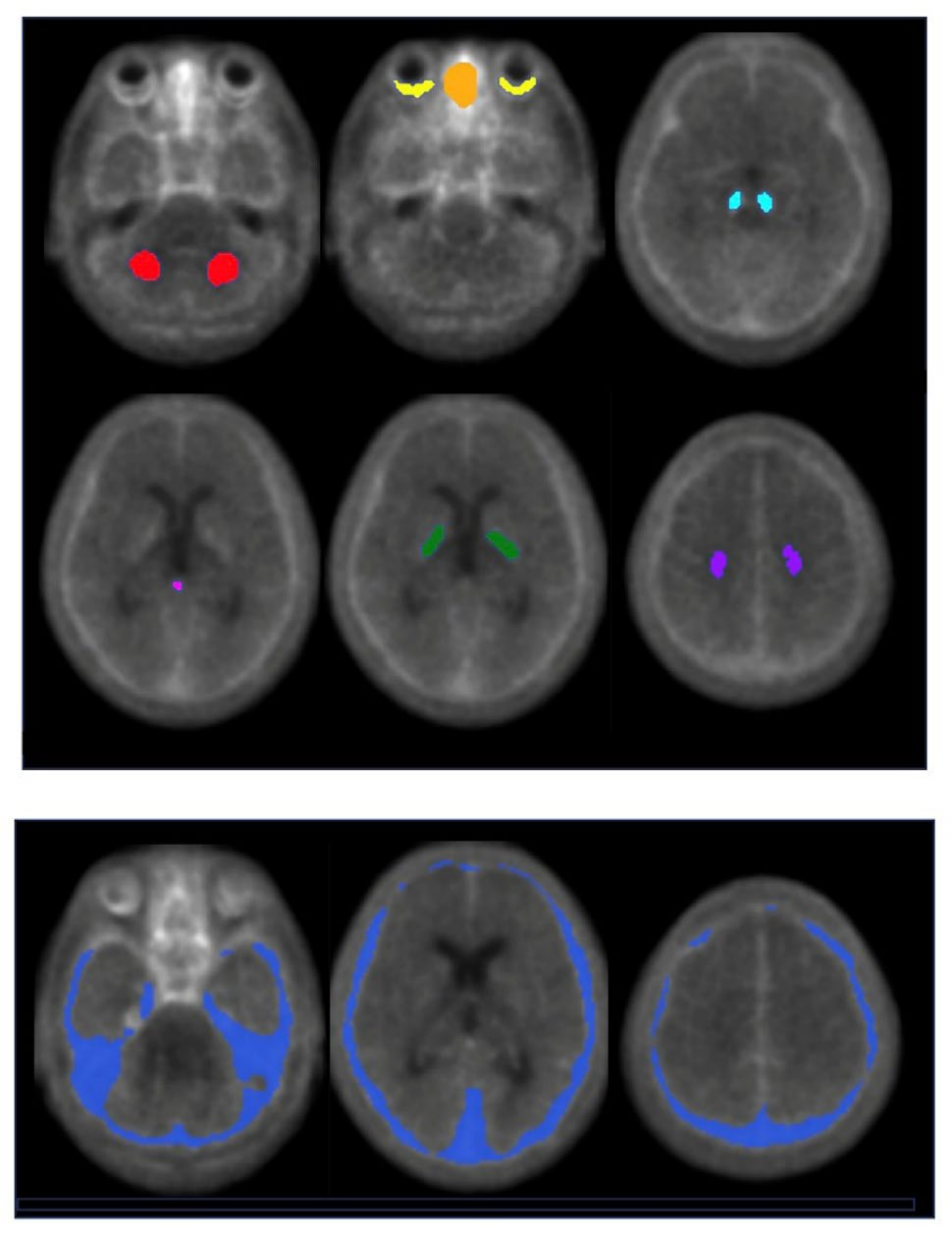
Regional reference volumes of interest (VOIs). *Red*: restricted cerebellar VOI, *yellow*: retina, *orange*: ethmoid, *moss green*: substantia nigra, *pink*: pineal gland, *green*: pallidum, *purple*: centrum semiovale, *blue*: meninges

As depicted in Fig. 1, the volume of interest (VOI) for the cerebellum were placed in the central region to avoid the influence of meningeal uptake as a restricted cerebellar VOI reference region (reCb) shrunken medially without the spill-in of meningeal accumulation in ^18^F-MK-6240 images. The ^18^F-MK-6240 images were normalized to cerebellar mean values using this reCb VOI. Next, VOIs for the substantia nigra (SN), centrum semiovale, pallidum, pineal gland, pons, meninges, retina, and ethmoid sinus were delineated on the anatomically normalized mean image of the tau-negative group. These VOIs were then applied to the SUVR images of individual subjects for the measurement of SUVR values. Subsequently, anatomically normalized mean images were generated for the tau-negative and for the tau-positive group to facilitate a comparative analysis.

**Fig. 2:**
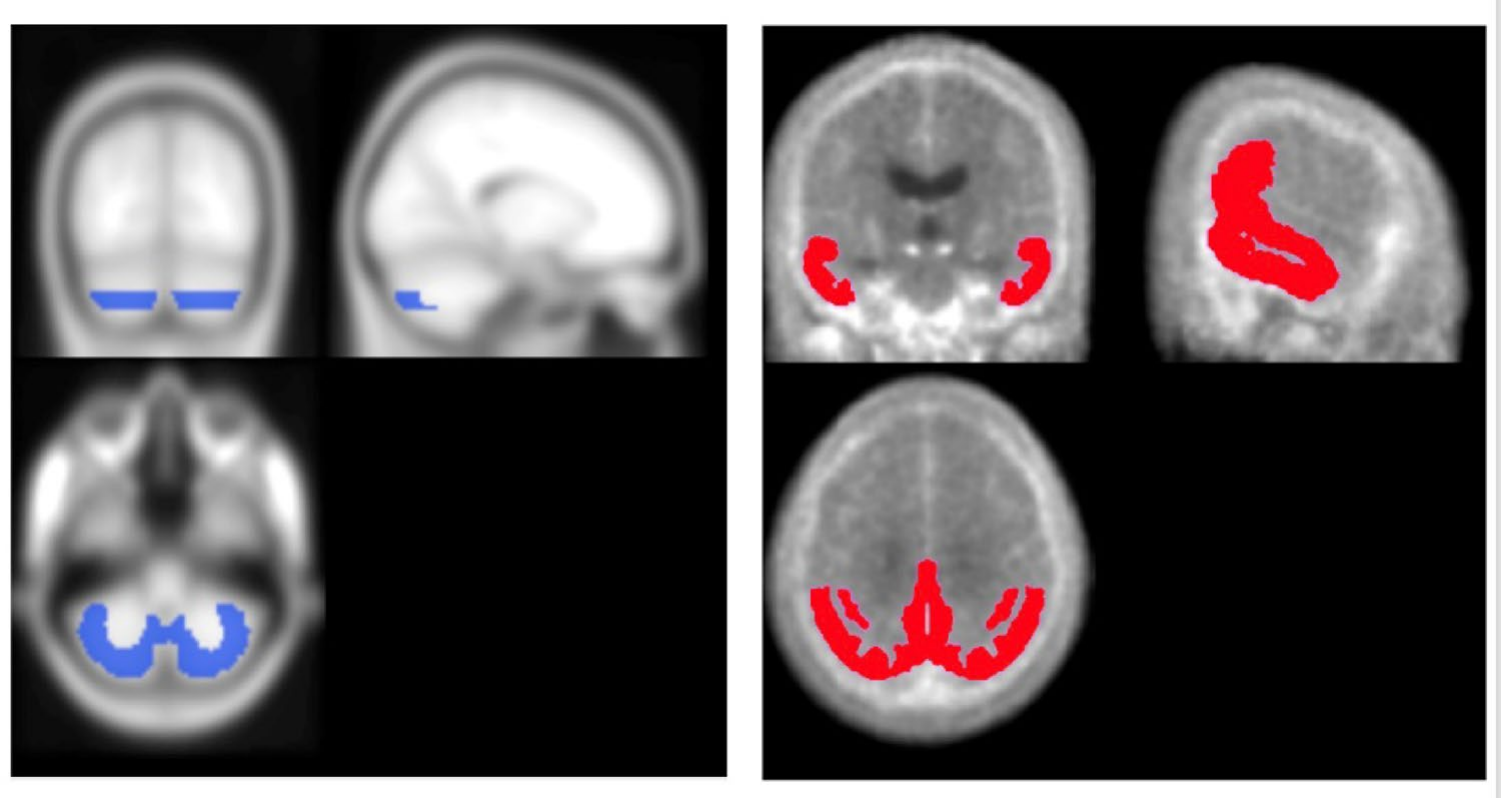
Reference VOIs for the CenTauR scale. *Blue*: cerebellar VOI, *red*: tau deposition target VOI

We also measured SUVRs using the VOIs for the CenTauR scale [19, 20] as shown in Fig. 2, and we evaluated the difference between the SUVR values obtained by using a reCb VOI and the SUVR values obtained by using the cerebellar gray matter VOI in CenTauR as the reference region.

### Statistical analysis

Group comparisons between the tau-positive and -negative groups were performed using independent-samples *t*-tests for each regional SUVR value normalized by the reCb. Multiple comparisons across brain regions were conducted using the Bonferroni correction. Regions with Bonferroni corrected p-values < 0.05 were considered significant. The differences in the SUVR between reCb VOI vs. CenTauR VOI were also evaluated. Given previous reports suggesting sex differences in meningeal uptake [4], we investigated whether there were any sex-related differences in the off-target accumulation across various brain regions.

We performed receiver operating characteristic (ROC) analyses to assess the diagnostic accuracy of the SUVRs using both reCb and CenTauR as reference regions to discriminate tau deposition that was positive or negative. The area under the curve (AUC) was calculated for each metric, and the statistical comparison between AUCs was conducted using the bootstrap method (10,000 resamples).

## Results

Nine tau-negative subjects and 26 tau-positive subjects were selected for this study. Table 1 summarizes the subjects’ demographic data. The tau-negative group was comprised of the following subjects: subjective cognitive decline (*n* = 1 subject), progressive supranuclear palsy (*n* = 1), suspected non-AD pathophysiology (*n* = 3), frontotemporal lobar degeneration (*n* = 2), and mild cognitive impairment (MCI) due to non-AD (*n* = 2). The tau-positive group was comprised of primary progressive aphasia (*n* = 1 subject), MCI due to non-AD (*n* = 4), MCI due to AD (*n* = 7), and AD (*n* = 14).

**Table 1:** Demographic data of the tau-negative and positive groups

	*n*	Sex	Age	MMSE
Negative	9	4 females, 5 males	77.0 ± 3.9	26.7 ± 4.2
Positive	26	19 females, 7 males	68.2 ± 10.8	23.8 ± 4.2

**Fig. 3:**
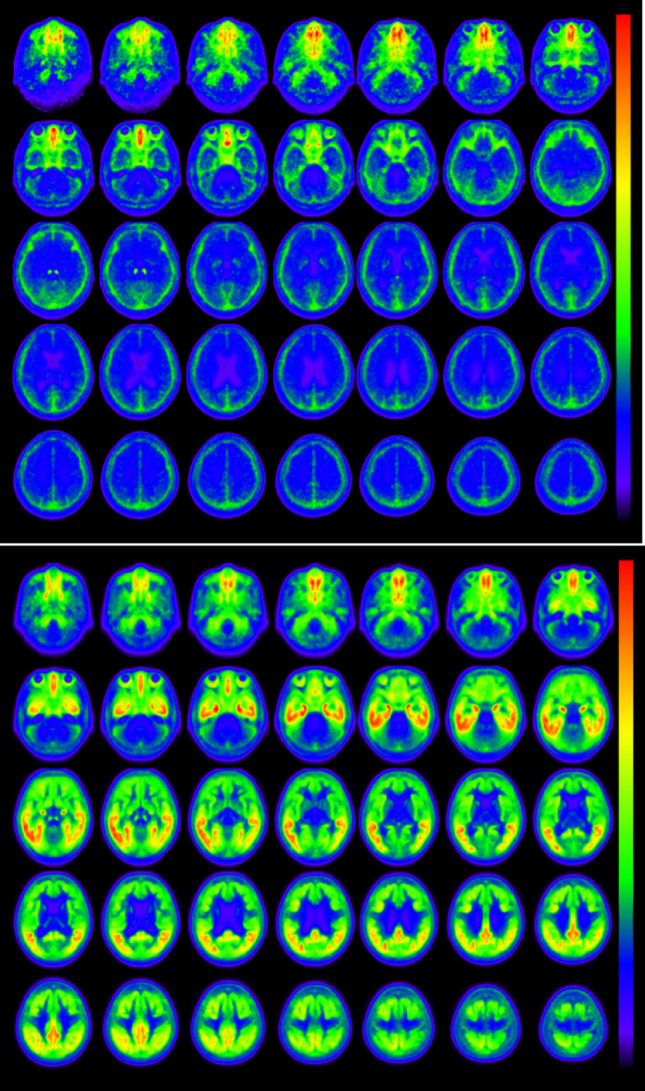
Mean ^18^F-MK-6240 images of the tau-negative and -positive subjects. **A**: Mean ^18^F-MK-6240 images of the tau-negative subjects (*n* = 9). **B**: Mean ^18^F-MK-6240 images of the tau-positive subjects (*n* = 26)

On visual assessment, intense off-target uptake was first observed in the ethmoid sinus, followed by the retina and meninges. Subsequent uptake was seen in the pallidum, substantia nigra, and pineal gland within the brain. In each subject, the uptake in the centrum semiovale was comparable to that in the cerebellum, and the uptake in the pons was noticeably lower than that in the cerebellum. Figure 3 provides the mean ^18^F-MK-6240 images of the tau-negative subjects and tau-positive subjects. Outside the cranium, intense radiotracer uptake was observed in the ethmoid sinus, and high uptake was also noted in the retina. Within the cranium, the meninges exhibited particularly high uptake, and additional accumulation was observed in the substantia nigra, globus pallidus, and pineal gland.

In the tau-negative group, the uptake in other brain parenchymal regions was minimal (Fig. 3A). In contrast, the tau-positive group demonstrated marked uptake in the lateral temporal cortex, medial temporal structures including the hippocampus and amygdala, parietal association areas, and the posterior cingulate cortex and precuneus, with additional involvement of other neocortical regions. Mild uptake was also observed in the striatum, while little to no uptake was seen in the primary sensorimotor cortex (Fig. 3B).

**Fig. 4:**
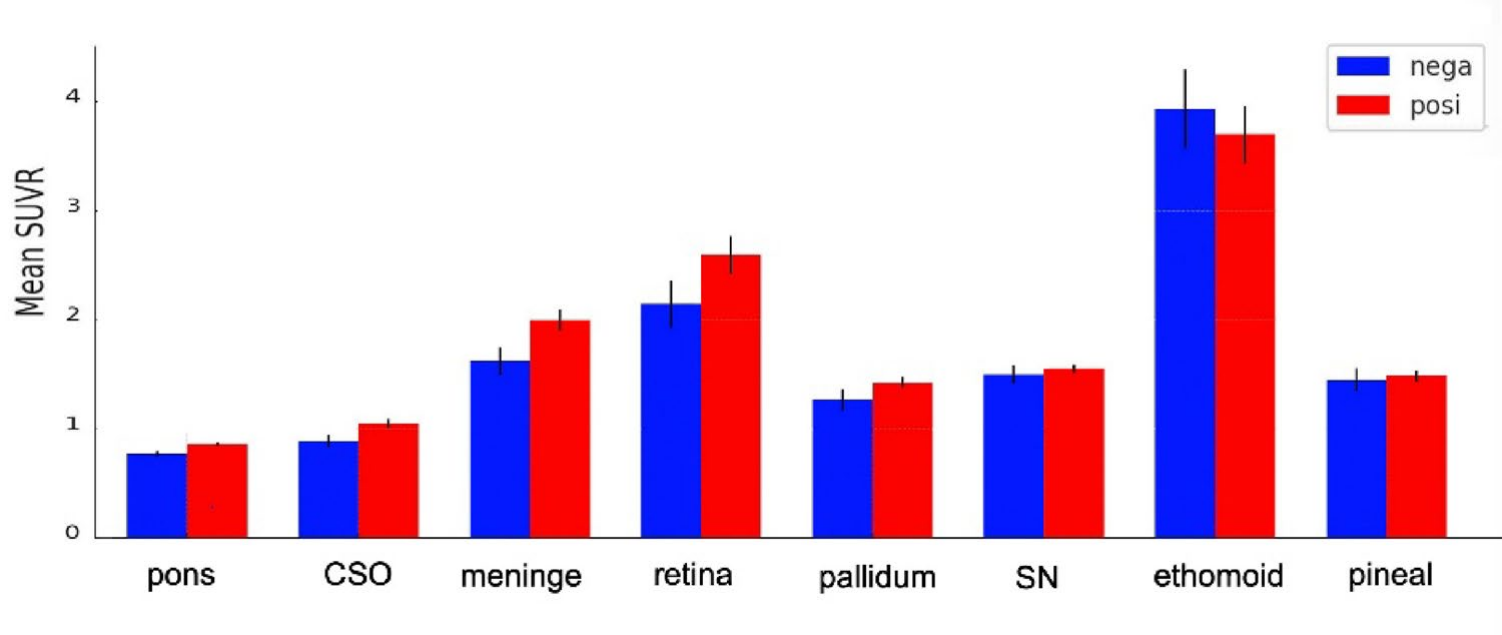
The mean SUVRs calculated using the restricted cerebellar VOIs of each region

There were significant group differences between the tau-negative and -positive groups in several regional SUVR values (Table 2; Fig. 4). Specifically, the pons, meninges, and centrum semiovale showed significant between-group differences at an uncorrected threshold of *p* < 0.05. However, after correction for multiple comparisons using Bonferroni correction, none of the regions remained significant at *p* < 0.05 (Table 3).

**Table 2:** Regional SUVR calculated by restricted cerebellar reference

Region	Tau-negative group	Tau-positive group
Pons/reCb	0.77 ± 0.07	0.86 ± 0.06*
Centrum semiovale/reCb	0.89 ± 0.17	1.05 ± 0.20*
Meninges/reCb	1.62 ± 0.38	2.00 ± 0.50*
Retina/reCb	2.15 ± 0.65	2.59 ± 0.89
Pallidum/reCb	1.27 ± 0.28	1.43 ± 0.23
Substantia nigra/reCb	1.50 ± 0.26	1.55 ± 0.19
Ethmoid sinus/reCb	3.93 ± 1.09	3.70 ± 1.34
Pineal gland/reCb	1.45 ± 0.32	1.49 ± 0.28

reCb: restricted cerebellar volume of interest

**Table 3:** Regional SUVRs calculated using the CentTauR cerebellar reference

Region	Tau-negative group	Tau-positive group
Pons/CenTauR	0.64 ± 0.13	0.72 ± 0.09
Centrum semiovale/CenTauR	0.75 ± 0.22	0.87 ± 0.20
Meninges/CenTauR	1.32 ± 0.28	1.66 ± 0.44
Retina/CenTauR	1.80 ± 0.69	2.13 ± 0.67
Pallidum/CenTauR	1.06 ± 0.32	1.19 ± 0.23
Substantia nigra/CenTauR	1.25 ± 0.33	1.29 ± 0.19
Ethmoid sinus/CenTauR	3.23 ± 0.94	3.05 ± 1.01
Pineal gland/CenTauR	1.20 ± 0.30	1.23 ± 0.21

**Fig. 5:**
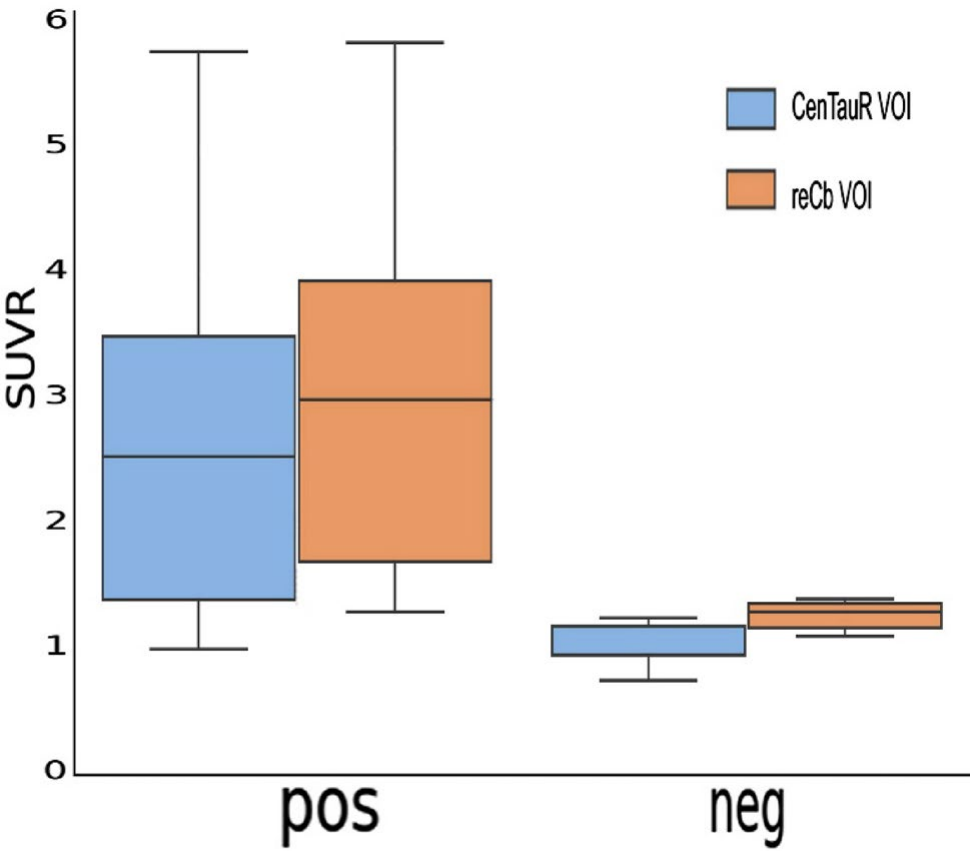
Comparison of the SUVRs calculated using the restricted cerebellar VOI and CenTauR cerebellar VOI in the tau-negative and tau-positive groups

Paired *t*-tests comparing the CenTauR SUVRs versus the reCb within each tau group demonstrated significant differences in both the tau-negative and -positive groups, with higher SUVRs observed when the reCb reference was used. The one-way ANOVA of these difference scores showed a significant group effect (F = 6.03, *p* = 0.019), suggesting a potential interaction between group and reference selection Fig 5.

Although a trend toward higher meningeal SUVR values in females compared with males was observed, this difference did not reach statistical significance (Tables 4 and 5).

**Table 4:** Regional SUVR difference between the females and males calculated using the restricted cerebellar reference (reCb)

Region	Females *n* = 23	Males *n* = 12
Pons/reCb	0.84 ± 0.07	0.81 ± 0.17
Central semiovale/reCb	1.00 ± 0.19	0.99 ± 0.27
Meninges/reCb	1.93 ± 0.33	1.85 ± 0.65
Retina/reCb	2.41 ± 0.87	2.40 ± 0.82
Pallidum/reCb	1.31 ± 0.20	1.41 ± 0.35
Substantia nigra/reCb	1.50 ± 0.16	1.53 ± 0.16
Ethmoid/reCb	3.89 ± 1.16	3.71 ± 1.46
Pineal gland/reCb	1.47 ± 0.28	1.41 ± 0.34

**Table 5:** Regional SUVR difference between the females and males calculated using the centaur cerebellar reference

Region	Females *n* = 23	Males *n* = 12
Pons/CenTauR	0.69 ± 0.11	0.70 ± 0.10
Central semiovale/CenTauR	0.83 ± 0.21	0.86 ± 0.21
Meninges/CenTauR	1.59 ± 0.32	1.52 ± 0.60
Retina/CenTauR	1.97 ± 0.67	2.18 ± 0.70
Pallidum/CenTauR	1.08 ± 0.22	1.29 ± 0.26
Substantia nigra/CenTauR	1.24 ± 0.21	1.36 ± 0.26
Ethmoid/CenTauR	3.19 ± 0.99	2.90 ± 0.98
Pineal gland/CenTauR	1.21 ± 0.23	1.25 ± 0.25

**Fig. 6:**
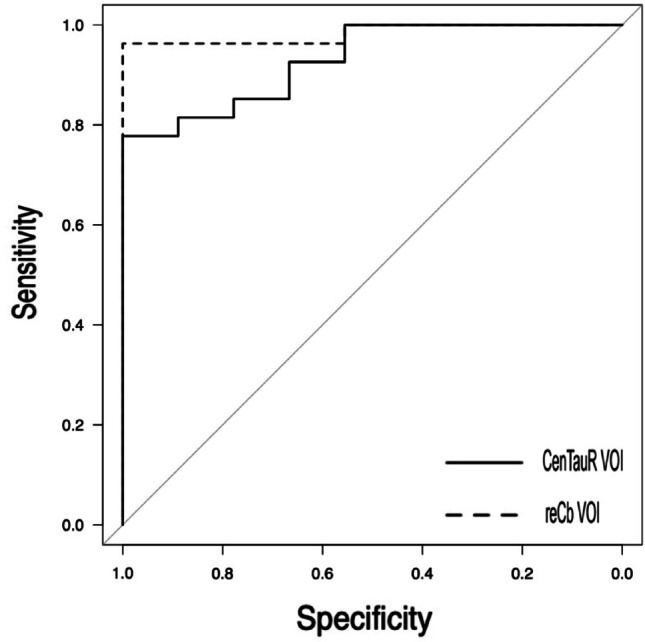
ROC analyses of the diagnostic accuracy for both reference methods

The results of the ROC analyses indicated high diagnostic accuracy for both reference methods, with AUCs of 0.930 for the CenTauR reference and 0.984 for the reCb reference (Fig. 6). Although the AUC for the reCb reference was numerically higher, the bootstrap comparison did not reveal a significant difference between the two curves (*p* = 0.109).

## Discussion

We evaluated the regional ^18^F-MK-6240 accumulations and the reference region sensitivity of ^18^F-MK-6240 tau PET using a high-resolution, brain-dedicated PET scanner. One of the major advantages of using a high-resolution PET scanner in this study was the improved spatial resolution, which allowed for more precise delineations of small anatomical structures and a better separation of the true parenchymal uptake from extracerebral signals [13].

The visual assessment in this study revealed off-target tracer uptake that was most prominent in extracranial structures such as the ethmoid sinus and retina. In contrast, the off-target uptake within the brain was minimal and often difficult to detect but was nevertheless identifiable in tau-negative individuals when the high-resolution scanner was used, even in averaged images with reduced resolution (Fig. 3). Off-target tracer accumulation in both normal and disease states complicates and confounds the interpretation of tracer binding in several subcortical regions. An off-target binding of ^18^F-MK-6240 in the retina and retinal pigment epithelium has been described as a characteristic off-target signal in both human imaging and autoradiographic postmortem studies [7, 21]. These findings highlight the importance of the careful interpretation of retinal signals in ^18^F-MK-6240 PET imaging, particularly when tau deposition near the orbit or optic pathways is being evaluated.

The midbrain uptake of a tracer in healthy elderly individuals may reflect binding to neuromelanin-containing cells in the substantia nigra [7–10]. The cause of off-target tracer accumulation in the globus pallidus, which is known to correlate with age and is independent of amyloid-β, remains unclear [22]. Regarding globus pallidus uptake, an increased accumulation in the globus pallidus has been observed in the majority of clinical phenotypes of four-repeat (4R) tauopathies including progressive supranuclear palsy (PSP), corticobasal syndrome (CBS), and corticobasal degeneration (CBD) with the use of [^18^F]PI-2620 tau PET, which may reflect underlying neurodegeneration. However, due to the variability in globus pallidus retention in healthy controls and AD, the visual identification of 4R tau pathology can sometimes be challenging [23].

Our analyses also revealed that the pons, meningeal regions, and centrum semiovale exhibited higher uptake in the tau-positive individuals. Although these results were not significant after Bonferroni correction, they suggest that certain brain regions may be sensitive to early tau deposition or reflect differences in anatomical patterns. The inclusion of white matter regions such as the centrum semiovale in the analyses adds to emerging literature suggesting that the tau PET signal in non-cortical areas warrants careful interpretation, especially when used for normalization or longitudinal tracking [11, 19].

In the present study, although a trend toward higher meningeal SUVR values in females compared with males was observed, a statistically significant difference was not detected, unlike the findings reported by Smith et al. [4]. This may be attributable to the smaller sample size in the present study compared with that of Smith et al., which may have limited the statistical power to detect a significant difference.

The advantage of better separation of true parenchymal uptake from extracerebral uptake was particularly evident in the cerebellum, where manually defined VOIs that excluded meningeal regions yielded significantly different SUVR values compared to the conventional cerebellar gray matter reference used in the CenTauR protocol. Our findings emphasize the methodological challenges posed by off-target accumulation and spill-in from extracerebral tissues, particularly in the meninges, pineal gland, and ethmoid sinus. These artifacts can confound quantitative analyses, especially when cerebellar reference regions that are susceptible to such contamination are used [4, 5]. This is particularly relevant in the calculation of SUVRs when cerebellar reference regions that are prone to contamination from extracerebral activity are used [11]. The statistically significant SUVR differences that we observed in both the present tau-positive and -negative groups underscore the critical importance of VOI selection in ^18^F-MK-6240 PET quantification.

The results of our ROC analysis demonstrated that both the standard and restricted cerebellar reference VOIs offer high diagnostic performance, with AUCs exceeding 0.93. Although the restricted VOI yielded a slightly higher AUC, this difference did not reach statistical significance. These findings are consistent with reports describing the excellent classification performance of ^18^F-MK-6240, even in early disease stages [2, 5]. Although spatial refinement improves quantification, it may not substantially alter diagnostic classifications. Although no statistically significant difference was observed in diagnostic (classification) performance, the reCb method is superior to the CenTauR method in terms of quantitative accuracy because it prevents underestimation of SUVR. Therefore, in longitudinal assessments of disease progression and therapeutic response, subtle changes that may not be detectable with the CenTauR method due to underestimation could potentially be detected using the reCb method, suggesting the potential utility of this approach [11, 20].

## Conclusion

Our findings underscore the importance of addressing off-target signal and reference region contamination in ^18^F-MK-6240 PET imaging. The use of a high-resolution PET scanner enabled clear visualization of fine anatomical structures on ^18^F-MK-6240 PET images. Future research should further evaluate these findings in larger cohorts and explore automated VOI definition strategies to facilitate standardization across clinical and research settings.

## Data Availability

The data supporting the findings of this study are available on request from the corresponding author.
